# Why (and how) should we study the interplay between emotional arousal, Theory of Mind, and inhibitory control to understand moral cognition?

**DOI:** 10.3758/s13423-016-1042-5

**Published:** 2016-05-11

**Authors:** Marine Buon, Ana Seara-Cardoso, Essi Viding

**Affiliations:** 1 Division of Psychology and Language Sciences, University College London, London, UK; 2 Neuropsychopysiology Lab, CIPsi, University of Minho, Braga, Portugal; 3 Institute of Cognitive Neuroscience, University College London, London, UK

**Keywords:** Morality, Dual processes model, Empathy, Theory of mind, Inhibitory control, Intention, Causation

## Abstract

Findings in the field of experimental psychology and cognitive neuroscience have shed new light on our understanding of the psychological and biological bases of morality. Although a lot of attention has been devoted to understanding the processes that underlie complex moral dilemmas, attempts to represent the way in which individuals generate moral judgments when processing basic harmful actions are rare. Here, we will outline a model of morality which proposes that the evaluation of basic harmful actions relies on complex interactions between emotional arousal, Theory of Mind (ToM) capacities, and inhibitory control resources. This model makes clear predictions regarding the cognitive processes underlying the development of and ability to generate moral judgments. We draw on data from developmental and cognitive psychology, cognitive neuroscience, and psychopathology research to evaluate the model and propose several conceptual and methodological improvements that are needed to further advance our understanding of moral cognition and its development.

## Introduction

Recent findings in the fields of cognitive psychology and cognitive neuroscience support the notion that morality is made up of multiple complex processes and implicate a widely distributed network of brain areas (Moll, Zahn, de Oliveira-souza, Krueger, & Grafman, 2005; Young & Dungan, 2011). Importantly, it is now clear that processes such as empathy (Reniers et al., 2012), Theory of Mind (ToM) (Young, Cushman, Hauser, & Saxe, 2007), executive control (Greene, Morelli, Lowenberg, Nystrom, & Cohen, 2008; Moore, Clark, & Kane, 2008) and abstract reasoning (Greene, Nystrom, Engell, Darley, & Cohen, 2004) are typically deployed when computing moral judgments and moral decisions.

**Fig. 1:**
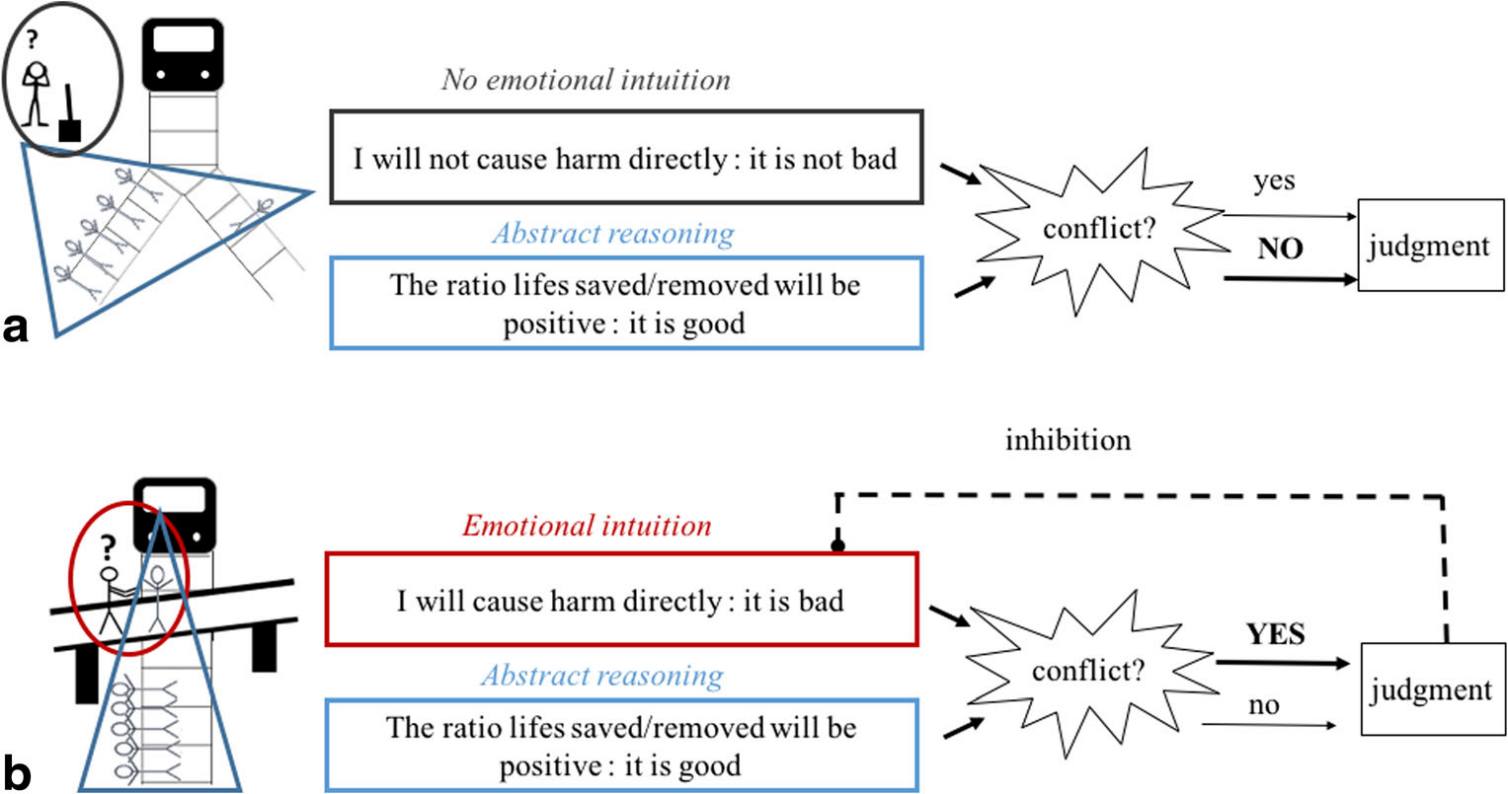
Schematic representation of Greene’s dual processes model of moral judgment. This figure describes the processes underlying individuals’ judgment about (**a**) the Trolley dilemma and (**b**) the Footbridge dillema

Building upon this recent evidence, several authors have theorized that morality is supported by distinct evaluative systems that can act in concert, in competition, or in conflict, each resting upon specific cognitive processes, and help individuals decide what is right and what is wrong (Cushman, Young, & Greene, 2007; Cushman, 2008; Greene, 2009). For instance, Greene’s dual process of morality (Greene, 2009) describes the computations engaged in the processing of the now famous Trolley dilemmas. In these type of dilemmas, a trolley is running out of control on a track and is about to kill five people. Individuals have to decide whether or not they would kill a man to save five people either by pulling a lever that will switch the trolley to a track where only one man is standing (Fig. 1a), or by pushing a man off a footbridge and into the path of the trolley in order to stop it (Fig. 1b). Typically, whereas individuals tend to say they would pull the lever to save five people (and thus give a utilitarian judgment, i.e., a judgment that focuses on the consequence of the action), most say they would not push the man to save the same number of people (and thus make a deontogical judgment, i.e., a judgment that focuses on the wrongness of the action itself). According to Greene’s model, generating an utilitarian judgment when faced with pushing someone off the footbridge is difficult because individuals undergo a strong cognitive conflict: on one side, a system responsible for the evaluation of the action to be performed generates an automatic emotional aversion that leads them to condemn the action; and on another side, a system responsible for the rational evaluation of the utilitarian consequences of the action (i.e., the ratio lives saved/deaths) leads them to consider this action as permissible. Critically, in order for this conflict to be solved in favor of the “rational” evaluation, people need to deploy inhibitory control to override the initial strong negative emotional aversion to pushing someone off the bridge. While there is strong evidence supporting this theoretical proposition (for review, see Cushman, Young & Greene, 2010), the explanatory power of this model remains restricted to judgment of highly complex and highly specific moral situations (situations in which deontological and utilitarian judgments are in conflict). This prevents the generalization of such an architecture of moral cognition to other moral situations where there may also be a conflict between emotion processing and other aspects of cognition, but which do not require an utilitarian computation. Furthermore, even thought the type of architecture proposed by Greene (2009) may have an important impact in the study of the development of moral competencies (Buon, Habib & Frey, 2015), trolley-like moral dilemmas are especially complex, which limits the investigation of this model in young populations using such dilemmas (but see Pellizzoni, Siegal & Surian, 2010).

Cushman’s (2008) dual-process model of morality also specifies an interplay between two different evaluative systems, one involving the evaluation of the agent’s causal responsibility (i.e., whether the agent caused harm), the other involving the evaluation of the agent’s mental state (i.e., whether the agent intended to cause harm), as critical for making moral judgments. The basic situations of harm that have been described to test the hypotheses set out by this model are present in our everyday life and different from the complex trolley and footbridge dilemmas. However, they still typically involve a set of different computations and likely require a complex interplay between the evaluative systems for causal responsibility and agent’s mental state, which can act either in concert, in conflict, or in competition. While Cushman, Sheketoff, Wharton, and Carey (2013) agrees that ToM competencies and executive control are probably useful in helping individuals integrate information about intention to their moral judgment, he does not provide a precise description of the way in which different processes may interact in adulthood and during the development to allow individuals to judge typical situations of harm. In addition, the potential affective nature of the processes underlying causal and intentional evaluations is not considered. Instead, Cushman’s dual-processes model focuses on the privileged information that different types of moral judgments (i.e., wrongness and punishment) may take as critical inputs. More specifically, for Cushman, whereas wrongness judgment tends to exclusively depend on information about intentions, punishment judgment primarily relies on information about causation, but is constrained by intent/wrongness judgment.

**Fig. 2:**
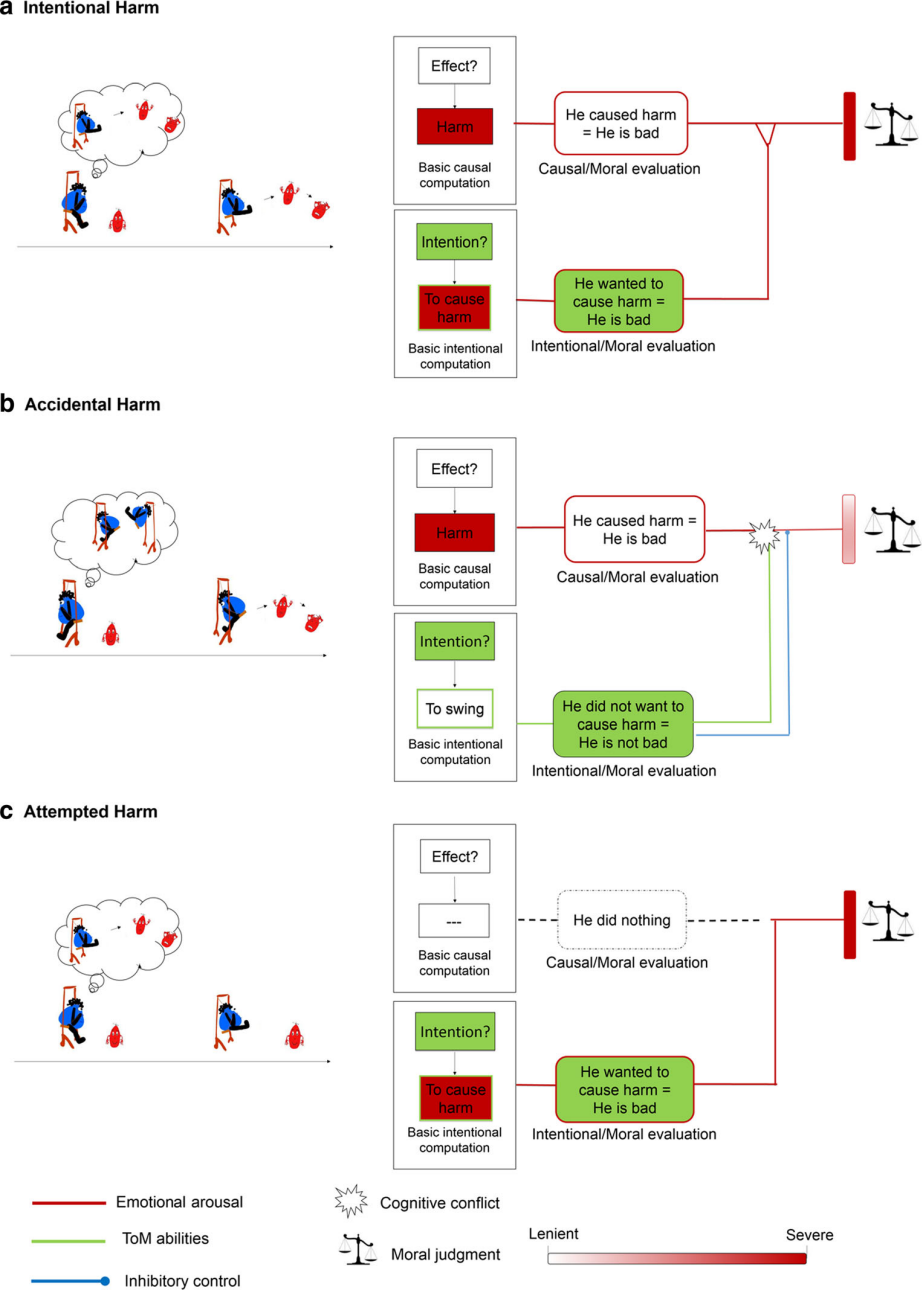
The ETIC (E=emotional arousal, T=theory of mind, IC=inhibitory control) model of morality. Schematic representation of the processes involved in moral judgment about agents who committed intentional harm, accidental harm, and attempted harm. (**a**) An intentional harm. Mr Blue is on a swing, sees Mr Red arriving and strikes him. His intention is negative (to strike Mr. Red) and his causal role is negative too (he strikes Mr. Red). (**b**) An accidental harm. Mr Blue is on a swing with his back to Mr Red and hits him accidentally. His intention is neutral (to swing) but his causal role is negative (he swings back and hits Mr Red). (**c**) An attempted harm. Mr Blue is on a swing, sees Mr Red arriving and tries to hit him. Mr Blue intention is negative (to hit Mr Red) but his causal role is neutral (he fails to hit Mr Red)

In the present article, we aim to provide an account of the cognitive-affective processes that underlie our ability to generate and integrate evaluations of an agent’s *causal role* and of the agent’s *intention* to cause harm in our moral judgments. More specifically, our goal is to characterize the way in which individuals successfully generate and integrate information about an agent’s causal responsibility, and his intention to harm, in moral judgments of harmful actions. Let's imagine a situation: If we see Mr Blue pushing Mr Red and injuring him, we are likely to get an automatic emotional response as we see Mr Red’s suffering (see Fig. 2a and b). This automatic response may lead us to evaluate Mr Blue badly. But, as adults, we are also likely to evaluate whether Mr Blue harmed Mr Red *intentionally* or not. If, due to our ability to represent mental states, we deduce that Mr Blue’s act was unintentional, we will likely exculpate him for his harmful behavior (Fig. 2b). We will thus inhibit the negative evaluation initially triggered by the perception of harm that was caused. Alternatively, if we think that Mr Blue’s act was intentional, we will assign him the maximum blame (Fig. 2a).

In the following sections, we will first detail a *working* model of processes underlying the ability to generate moral judgments of harmful actions in which emotional arousal, ToM capacities, and inhibitory control resources have a critical role (section The ETIC model of morality). This model, that we will call the ETIC (E=emotional arousal, T=theory of mind, IC=inhibitory control) model of morality, will serve as a framework for critically reviewing and discussing findings from cognitive psychology and neuroscience (section Cognitive and neural architecture underlying moral judgment about harmful situations: Insights from adult samples), as well as from developmental psychology (section Moral judgement in typically developing children) and psychopathology (section Insights from clinical populations), that have a bearing on the model. By doing so, we hope to contribute to the quickly growing literature in several ways. First, the ETIC model is the first attempt to bind and advance Greene’s and Cushman’s important theoretical propositions into a comprehensive account of the processes involved in our judgments of basic harmful actions. The ETIC model assumes that the core of morality is grounded in an emotional intuition triggered by the perception of a harmful action, which is subsequently modulated or inhibited by higher evaluative systems, and is therefore structurally similar to Greene’s proposition. Analyzing its empirical validity is critical to understand whether our moral competencies rely on a generic cognitive architecture which allows us to judge different types of morally salient situations. Second, by systematically investigating the precise computations involved in processing basic harmful actions, our critical review will shed light on several issues that need to be addressed if we want to refine our understanding of the role of, and the interaction between, emotional processes (section The role of affective processing in integrating information about the agent’s causal role and intention to harm into moral judgment), ToM (section The role and nature of ToM mechanisms in moral judgments), and inhibitory control (section Exploring the contribution of inhibitory control resources) in both moral judgment and moral behavior (6.3) from a life-long and multidisciplinary perceptive.

## The ETIC model of morality

What computations are employed when we make a moral evaluation about Mr Blue’s behavior (Mr Blue pushes Mr Red, Fig. 2)? We may employ a system responsible for the evaluation of the causal properties of the agent's action, and a system responsible for the evaluation of the agent’s intention (Cushman, 2008). The first system would be composed of basic computations of the agent’s causal role, while the latter of computations of the intentional state of the agent. In each case, these computations would be followed by moral evaluations of the agent’s causal role and intention to harm; each moral evaluation providing a separate valenced response from a moral standpoint (causal-moral evaluation: he caused harm = he is bad; intentional-moral evaluation: he wanted to cause harm = he is bad). This dual-processes account of moral judgment abilities has several implications. Notably, it proposes – in line with Cushman’s model – that the two moral evaluations could act in concert, in conflict, or by entering in competition. For instance, in case of *intentional harm* (see Table 1, when someone causes negative outcome with negative intention), one could expect the two moral evaluative systems to generally act in concert because of their congruent outputs. *Accidental harm* (when someone causes negative outcome with neutral or positive intention that)*,* in contrast, should generally trigger a cognitive conflict given the opposite outputs arising from the causal-moral evaluation and the intentional-moral evaluation (he caused harm = he is bad; but he did not want to cause harm = he is not bad). Finally, in case of *attempted harm* (when someone has negative intention but causes nothing), one should expect the two moral evaluative systems to act competitively. If the agents’ action causes no harm at all, the causal-moral evaluation system would not be engaged and the intentional-moral evaluation would dominate the moral judgment.

**Table 1:** Outputs from causal/moral and intentional/moral evaluations depending on the situation perceived

Situation/evaluation	Causal/moral evaluation	Intentional/moral evaluation
Intentional Harm (negative intention and negative outcome)	He caused harm = he is bad	He wanted to cause harm = he is bad
Accidental Harm (neutral or positive intention and negative outcome)	He caused harm = he is bad	He did not want to cause harm = he is not bad
Attempted Harm (negative intention and no or neutral outcomes)	He caused nothing	He wanted to cause harm = he is bad

The consideration of the computations implicated in each type of evaluation leads us to question the potential role of the three cognitive processes in moral judgment: emotional arousal, ToM capacities, and inhibitory control resources. Emotional arousal, operationalized here as the affective response that is automatically triggered by the observation or the representation of someone else’s distress (Eisenberg, 2000; Seara-Cardoso, Neumann, Roiser, Mccrory, & Viding, 2012), is proposed to be critical for generating an automatic and negative reaction to Mr Red (the victim)’s distress. ToM capacities, defined as the ability to represent and use agents’ mental states to explain other people’s behaviors (Premack & Woodruff, 1978), is proposed to be relevant for basic computations of the agent’s intention as well as to morally evaluating them. Finally, inhibitory control resources, defined here as those cognitive processes that enable regulation and control of other cognitive processes (Chan, Shum, Toulopoulou, & Chen., 2008), might be specifically useful for solving conflicts if the outputs from the two evaluative systems are incongruent (e.g., in case of *accidental harm*). We will illustrate further the involvement of these processes by analysing each type of harmful situation in turn.

In the case of *intentional harm* (Fig. 2a), we propose that individuals will typically generate a moral judgment as follows: First, an automatic negative emotional reaction to Mr Red’s suffering/distress is likely to occur through basic emotional arousal triggered by the emotional contagion taking place when one perceives someone else’s distress (see Decety & Cowell, 2014). This reaction is likely to be linked to Mr Blue’s through a basic computation of physical causality, i.e., who caused the harm (basic causal computation). The automatic negative response would thus be the critical input for the causal-moral evaluation of the agent (he caused harm = he is bad). In parallel, the computation of Mr Blue’s intention would engage ToM capacities that would allow us to determine what he wanted to do. Here, a negative emotional reaction is likely to arise from the representation of Mr Blue’s intention (he wanted to push = he is bad). This negative emotional reaction is either sustained or revised during the moral-intentional evaluation that aims to assess, through additional ToM computations, the agent’s ultimate intentions from a moral standpoint (he wanted to push to cause harm = he is bad). That is, one would bring together the initial intentional computation and other relevant contextual information to understand the very nature of the agent’s intention (for instance Mr Blue might have wanted to push Mr Red because he wanted to cause harm or because he wanted to avoid Mr Red being hit by a car). Finally, the integration of these causal-moral and intentional-moral evaluations in the final moral judgment should be relatively straightforward in the case of intentional harm. The outputs of the different evaluative systems are of the same value (cause = he is bad, intention = he is bad), and should thus reinforce each other and lead us to evaluate Mr Blue negatively.

In the case of *accidental harm* (Fig. 2b), we propose that individuals typically generate a moral judgment as follows. When Mr Blue commits *accidental harm*, the two first steps involved in basic causal computation and causal-moral evaluations should be the same as the ones involved in the *intentional harm* scenario described above and should thus give rise to a negative output (cause = he is bad). Basic intentional computation should also involve ToM capacities but should result in a neutral output (e.g., Mr Blue wanted to swing). From a moral standpoint, the intentional-moral evaluation should also lead to neutral output (he did not want to cause harm = he is not bad), which would then conflict with the negative output arising from the causal-moral evaluation. Here we would need to deploy inhibitory control resources to inhibit the negative evaluation arising from the causal-moral evaluation. Importantly, ToM computations should be maintained during this process to provide the competing output (he is not bad) that is prioritized over the output arising from the causal-moral evaluation (he is bad). To summarize, we propose that moral judgment of *accidental harm* arises from the interplay between the negative emotional arousal elicited by the victim’s suffering, ToM computations regarding the Mr Blue’s intentions, and the deployment of inhibitory control, which would enable the integration of the agent’s neutral intention in the final moral evaluation.

In the case of *attempted harm* (Fig. 2c), we propose that individuals typically generate a moral judgment as follows. In contrast to the previous situations described, the basic causal computation should yield nothing since the agent’s action does not cause harm. The causal-moral evaluation should thus remain dormant. However, the computations of the agent’s intention by deployment of ToM capacities should generate a negative emotional reaction via the emotional arousal elicited by the representation of the agent’s intention to cause harm and thus the outcome desired by the agent. It should be noted that in this case, individuals’ negative emotional reactions do not rely on a basic mechanism of emotional contagion as in the case of basic causal computation. Indeed, without overt emotional cues, individuals’ affective reaction should rely on individuals’ ability to infer what the other person is feeling based on various non-emotional cues, by putting oneself in the other’s place (i.e., affective perspective taking; Eisenberg, Shea, Carlo, & Knight, 1991, see also Vaish, Carpenter & Tomasello, 2009).

Like in the case of judgment of *intentional harm*, this negative emotional output is either sustained or revised through additional ToM computations to morally evaluate the agent’s intention (he wanted to push to cause harm = he is bad). Here, there is no conflict with the output arising from the causal evaluation (since the causal-moral evaluation is not triggered). In this case, inhibitory control resources would not be necessary and the final moral judgment should depend on the intentional-moral evaluation alone (he is bad). To summarize, moral judgment of *attempted harm* should mainly rely on ToM capacities and emotional arousal resulting from ToM computations.

In the ETIC model, we make several assumptions regarding the cognitive architecture underlying the processing of harmful situations that need to be examined in the light of the existing literature. Notably, the model proposes that causal-moral and intentional-moral evaluations are achieved by distinct computations and that emotional arousal, ToM capacities, and inhibitory control resources impact differently on moral judgments about *intentional harm*, *accidental harm,* and *attempted harm*. In contrast to Greene’s initial proposition (Greene et al., 2004; Greene, 2009), the ETIC model assumes that affective computations are not restricted to one type of evaluation but are instead required for both the evaluation of the agent’s causal role and of the agent’s intentions (Shenhav & Greene, 2014). Importantly, the ETIC also differs from Cushman’s model (Cushman, 2008; Cushman et al., 2013) by being agnostic about the relative weight given to information about intention and causation in judgments of wrongness and punishment judgments.

In its current form, the ETIC model thus predicts that the interaction between emotional arousal, ToM capacities, and inhibitory control should be the same regardless of whether an individual is asked to judge the wrongness of the action or whether the action should result in a punishment (see Table 2, for comprehensive predictions regarding Cushman and the ETIC models). However, since, very few studies have systematically compared different types of moral judgments (but see Cushman, 2008; Cushman et al., 2013), it was not possible to systematically explore the validity of those specific predictions in our critical review. Furthermore, as will be discussed further in section Moral judgement in typically developing children, the ETIC model and Cushman’s model are not mutually exclusive.

**Table 2:** Predictions made by the ETIC (E=emotional arousal, T=theory of mind, IC=inhibitory control) model and Cushman’s model of moral judgment (based on Cushman, 2008; Cushman et al., 2013)

	Cushman’s model	ETIC model
Common claims	• Causal and intentional evaluations are underlied by distinct psychological systems. • Causal and intentional evaluation can act in concert, in conflict or in competition.	
Main assumptions and predictions	• Judgment of wrongness based on the mental state that determined the action • Judgment of punishment based on causal responsibility for harm and constrained by judgment of intent/wrongness → punishment judgment is more influenced by causal responsibility than by the agent’s intention to harm, as compared with wrongness judgment • ToM capacities and inhibitory control resources probably help (but are not sufficient) to generate intent-based moral judgment of attempted and accidental harms	• The integration of the agent’s causal responsibility into moral judgment depends on emotional arousal • The integration of the agent’s harmful intention into moral judgment depends on the interaction between emotional arousal, ToM capacities • The integration of the agent’s innocent intention into moral judgment depends on the interaction between emotional arousal, ToM capacities and inhibitory control resources
Developmental predictions	• Intent-based wrongness judgment mostly depends on children’s conceptual achievements • Intent-based judgment would first emerge for wrongness judgment, then for punishment judgment	• Children develop the ability to generate intent-based moral judgment for attempted harm, alongside the developement of ToM capacities • Generation of intent-based moral judgment for accidental harm develops later, with the the development of inhibitory control resources
Individuals with ASD	No clear predictions	• Given ToM difficulties, individuals with ASD differ from control participants when judging attempted harm (less severely) and of accidental harm (more severely)
Individuals with psychopathy/alexithymia	No clear predictions	• Given emotional difficulies, individuals with psychopathy/alexithymia differ from control participants when judging attempted harm (less severely) and accidental harm (less severely)
Individuals with ADHD	No clear predictions	• Given inhibitory control difficulties, individuals with ADHD differ from control participants when judging accidental harm (more severely)

*ToM* Theory of Mind, *ASD* autism spectrum disorder, *ADHD* attention-deficity hyperactivity disorder

In the next section, we will address the validity of the ETIC model by examining recent insights from cognitive psychology and cognitive neuroscience regarding the cognitive computations and brain areas implicated in adults’ processing of harmful situations. We will focus on the extant evidence regarding the role of emotional arousal, ToM capacities, and inhibitory control resources during moral judgments of *accidental* and *attempted harm* as these allow us to dissect the cognitive processes required for causal-moral and intentional-moral evaluations. We will not discuss situations involving *intentional harm* as such situations do not enable the separation of the cognitive processes required for causal-moral and intentional-moral evaluations (as both causal-moral and intentional-moral systems lead to the same output; see Cushman, 2008). We will then evaluate the model with findings from specific populations of interest: typically developing children, individuals with psychopathy, individuals with high levels of alexithymia, and individuals with autism. Finally, we will outline what critical phenomena need to be investigated further and propose several conceptual and methodological improvements that we think are required to (i) validate current models of moral cognition in a more comprehensive way; and (ii) yield a more complete picture of the cognitive processes underlying moral judgment about basic harmful action and moral behavior*.*

## Cognitive and neural architecture underlying moral judgment about harmful situations: Insights from adult samples

### What processes underlie moral judgments of *accidental harm*?

A recent study has investigated whether the integration of the agent’s causal role and of the intention to harm were similar in terms of their cognitive cost (Buon, Jacob, Loissel, & Dupoux, 2013). In this study, adult participants made moral judgments about agents who differed in their causal responsibility in a victim’s suffering (one agent caused harm accidentally while the other did not cause harm, but the victim hurt him/herself; i.e., the conditions were matched for distress cues) and about agents who differ on their intention to cause harm (one agent caused harm intentionally while the other caused harm accidentally). Critically, participants had to perform the task either under normal conditions or while engaging in a verbal shadowing task, which substantially taxes verbal and executive resources. When performing the task under normal conditions, participants were not only able to integrate both the agent’s causal responsibility and intention to harm in their moral judgment, but also preferentially weighted the agents’ intention. That is, participants were able to appropriately judge *accidental harm*, exculpating the agent when he had no intention to harm. This finding is in line with previous research (Cushman, 2008; Piaget, 1932; Young et al., 2007) and indicates that when judging *accidental harm*, adult participants give priority to the agent’s innocent intention instead of the agent’s causal role. In contrast, when participants judged *accidental harm* under verbal shadowing, although they were able to integrate the agent’s causal role in their moral judgment and were able to compute the agent’s intentions, they seemed unable to integrate the agent’s innocent intentions in their moral judgment. That is, when processing cases of *accidental harm* with diminished verbal and executive control resources, participants prioritized the agent’s causal role instead of the agent’s innocent intention in their moral judgments and did not exculpate the agent. This study supports the ETIC model, indicating that causal-moral and intentional-moral evaluations rely on two separable cognitive systems, which appear asymmetric in the degree to which they require verbal and/or executive resources to be available to integrate the causal and intentional evaluation outputs into a final moral judgment.

According to the ETIC model, while emotional arousal is a critical input to causal-moral evaluations, ToM capacities and inhibitory control resources are necessary to generate intentional-moral evaluations and to integrate the agent’s innocent intentions into moral judgment. In favor of this proposal, neuroscientific evidence demonstrates that processing accidental harm recruits brain regions typically involved in affective processing (e.g., amydgala), ToM computations (e.g., temporo-parietal junction), and conflict processing/regulation (e.g., Anterior Cingulate Cortex). For example, functional magnetic resonance imaging (fMRI) studies have demonstrated that when healthy adults judge accidental harm (compared to intentional harm), there is an increased neural response in the right temporo-parietal junction (rTPJ) and in the right temporal pole, regions that have been consistently associated with reasoning about others’ mental states (Berthoz, Grèzes, Armony, Passingham, & Dolan, 2006). Moreover, individuals who present higher rTPJ responses during moral judgment of accidental harm assign less blame to agents who cause harm accidently (Young & Saxe, 2009). Although correlational, these findings support the view that the neural circuitry typically engaged during ToM processing is deployed during moral judgments of accidental harm and is important for the integration of information of the agent’s innocent intentions.

With regard to the importance of inhibitory control resources, one fMRI study has reported that during moral judgment of accidental harm (compared to intentional harm) participants presented a pattern of brain responses (i.e., increased recruitment of right inferior parietal cortex, precuneus, bilateral anterior cingulate gyrus) similar to the one observed by Greene et al. (2004) during “high conflict moral dilemmas” which might be suggestive of a higher cognitive conflict during moral judgment of accidental harm (Young et al., 2007). Supporting this interpretation, several studies have reported individuals to take substantially more time to judge an agent for his accidental harm compared to someone who committed intentional harm or attempted harm (Buckholtz et al., 2008; Decety & Cacioppo, 2012; Decety, Michalska, & Kinzler, 2011; Imamoğlu, 1975; Young et al., 2007; Young & Saxe, 2009). A recent study also provides evidence in favor of the importance of regulatory mechanisms when judging accidental harm (Treadway et al., 2014). In this fMRI study, the authors observed that when participants judged accidental harm, the dorsal anterior cingulate cortex (dACC) exhibited top-down connectivity with the amydgala. This suggests thus that the dACC may have a critical role in regulating brain regions that are involved in affective computations (Treadway et al., 2014). Interestingly, additional connectivity analysis revealed that when viewing accidental harm, the dACC received greater input from the TPJ, which suggests that this top-down dACC regulation would rest upon participants’ ToM inferences regarding the agents’ mental states.

While encouraging and in line with the ETIC model, it should be noted that systematic and independent behavioral assessments of the participants’ emotional reactivity to other suffering, ToM and inhibitory control skills are necessary to confirm this interpretation of those neuro-functional data.

### What processes underlie judgment of *attempted harm*?

With respect to *attempted harm*, one experimental study has demonstrated that judgment of *attempted harm* generated a competition^1^ between causal evaluation and intentional evaluation (Cushman, 2008), which is in line with both the ETIC and Cushman’s model. Indeed, when processing attempted harm, and in contrast to cases of accidental harm, only one system is busy, which prevents a conflict from occuring. In this study, the author has reported significant differences in judgments of deserved blame and punishment of someone who fails to cause an *intentional harm* that does not occur, as compared with judgments of someone who fails to cause an *intentional harm* that does occur by some independent means. When an agent commits a failed attempt but the harm occurs by some independent means, causal responsibility is assigned to the independent means, and assessment of the culpable mental state of the agent is weighted less in the assignment of blame. By contrast, when no harm at all occurs, causal responsibility cannot be assigned. In these cases, an assessment of mental culpability dominates, leading to more severe judgments of punishment and blame with regard to the agent – compared with the condition where harm occurs by independent means. This “blame blocking” phenomenon demonstrates that, in case of attempted harm, causal and intentional moral evaluations are acting competitively to establish the moral judgment, which strongly argues in favor of the idea that causal and intentional evaluative systems rest upon separable systems acting competitively.

In line with our model, neuroimaging studies seem to indicate that brain regions that subserve affective processing and ToM reasoning are also critical for appropriate moral judgment of attempted harm. For example, Young et al. (2007) observed that the rTPJ, a region typically engaged during ToM tasks, is highly responsive during processing of attempted harm (contrasted to intentional harm; Young et al., 2007). Moreover, transcranial magnetic stimulation (TMS) applied on the rTPJ appears to reduce participants’ ability to blame an agent for attempted harm (Young, Camprodon, Hauser, Pascual-Leone, & Saxe, 2010). These findings suggest that the rTPJ, a region that subserves ToM computations, is critical for the proper evaluation of intention during moral judgment of attempted harm (Young, Camprodon, Hauser, Pascual-Leone, & Saxe, 2010).

Activity in the ventromedial prefrontal cortex (vmPFC), a brain area involved in ToM and value computations (Young & Saxe, 2009), has also been associated with the ability to blame an agent for attempted harm*.* The critical importance of this brain region in judgment of attempted harm has also been shown in two studies with patients with vmPFC lesions acquired in adulthood (Ciaramelli, Braghittoni, & di Pellegrino, 2012; Young, Bechara, Tranel, Damasio, & Hauser, 2010). In both studies, vmPFC-lesion patients were *less* likely than controls to blame an agent for attempted harm (i.e., when presented with attempted harm, vmPFC patients were less likely to integrate agents’ harmful intentions in their moral judgment). Ciaramelli et al. (2012) suggested that inefficient ToM computations might explain the atypical pattern of moral judgment in this population. However, the failure to consider the agents’ intention was not seen when intentions were neutral (i.e., in the case of *accidental harm*), which suggests that ToM computations that also involve affective valuation processes are selectively disrupted in vmPFC patients. Young and collaborators (2010) have suggested that the vmPFC may be critical for generating an emotional response from an abstract representation (see also Sobhani & Bechara, 2012). In other words, the vmPFC would be critical for negatively “coloring” the representation of the agent’s intent and this would be an important input for computation of the agent’s blameworthiness.

These data are in line with the model, suggesting that both intact ToM capacities and affective responses are required to blame an agent for attempted harm. However, additional studies are required to elucidate at what point ToM processes are employed in moral judgment. That is, to elucidate whether ToM processes are only deployed for making the basic intentional computations or are also critical for making intentional-moral evaluations.

### Summary: Insights from studies of adult samples

The extant evidence reviewed so far is in line with the model we have presented and suggests that: (i) separable systems are responsible for the causal-moral and intentional-moral evaluations in moral judgment; (ii) brain areas that have been consistently associated with affective, ToM, and inhibitory control processes are involved in moral judgment of harmful situations; but (iii) the involvement of these different brain regions differs as a function of the situation under evaluation.

While encouraging and in favor of our model and of the implication of affective, ToM, and inhibitory control processes in moral judgment, some caution needs to be exerted when interpreting neuroimaging data (Poldrack, 2006). For instance, even though the role of the vmPFC in moral judgment is well recognized, and most of the authors concur that the vmPFC is important for affective computations, the precise nature of these affective computations in moral judgment remains to be established. It is possible that the vmPFC plays a critical role the generation of emotional responses from abstracts representations (Young et al. 2010), but it is also plausible that it contributes to moral judgment by bringing affective valuations to decision-making processes (Shenhav & Greene, 2014) or through affective regulation (Ciaramelli, Braghittoni and di Pellegrino, 2012). Furthermore, some authors have also highlighted possible non-affective contributions that the vmPFC might make to moral cognition (e.g., self-projection; Ciaramelli & di Pellegrino, 2011) (see Mitchell, 2009 for a similar point with respect to the rTPJ). The fMRI data reported above thus may be insufficient to precisely demonstrate which computations (but also, at what point of the information processing chain) are critical when making moral judgments of accidental and attempted harm.

In the following section we will review studies that have focused on moral judgment during different periods of typical development. Emotional arousal, ToM, and inhibitory control capacities undergo substantial development throughout childhood and adolescence and this may help explain developmental differences in moral judgment and clarify the role of these different processes in moral judgment.

## Moral judgement in typically developing children

Emotional response to others’ distress, ToM, and inhibitory control capacities undergo substantive changes throughout childhood and adolescence. Developmental studies can thus provide a unique opportunity to assess the interplay of the different components of the ETIC model, in ways that are not possible in adults when all these components are fully mature and operational (de Haan & Gunnar, 2009).

To provide context, we will briefly examine the developmental course of the processes that we hypothesize to be at work in moral judgment computations.

The capacity for vicariously sharing the affective state of another emerges early during development. Early signs of emotional arousal in response to others’ suffering can already be seen in newborns, and it has been shown that infants become vicariously distressed when another infant begins to cry (Dondi, Simion, & Caltran, 1999). Even though the very nature of those affective reactions remains highly debated (Davidov, Zahn-Waxler, Roth-Hanania, & Knafo, 2013; Heiphetz & Young, 2014), this indicates that, by an early age, infants may show sensitivity to the negative nature of harmful outcomes, when perceive harmful interactions. Furthermore, before 1 year of age, evidence indicates that infants are able to track the causal structure of an event that modifies the physical state of an object (Muentener & Carey, 2010). It suggests that preverbal infants are not only able to track the harmful outcomes of an agent’s action but may also be sensitive to the causal relationship linking an agent and its (negative) impact on another’s emotional state. With age, reactions to others’ suffering become increasingly sophisticated. Toddlers begin to be able to distinguish between different emotional expressions and, later on, to label others’ emotions (Walle & Campos, 2012). Around the age of 2 years, with the development of the ability to distinguish the self from others, children begin to be able to differentiate between their own from others’ internal states, and start displaying behavioral expression of empathic concern (i.e., helping / comforting behaviors) (Decety, 2010; Roth-Hanania, Davidov, & Zahn-Waxler, 2011). Importantly, at about the same age, toddlers seem to be able to express empathic concern towards victims of harmful actions who do not express overt emotional expressions (Vaish, Carpenter, & Tomasello, 2009).

Regarding the development of ToM capacities, even though there are studies suggesting that preverbal infants display some sensitivity to others’ mental states such as others’ goals, beliefs, and false beliefs (Baillargeon, Scott, & He, 2010; Onishi & Baillargeon, 2005), it seems that ToM capacities such as the ability to infer desires, intentions, beliefs, and false beliefs are typically acquired and expressed between the age of 3 and 5 years (Wellman, Cross, & Watson, 2001). However, it is important to keep in mind that flexible deployment of ToM, as well as more sophisticated ToM reasoning, continue to develop until mid-adolescence (Apperly, Warren, Andrews, Grant, & Todd, 2011; Dumontheil, Apperly, & Blakemore, 2010).

Finally, the development of inhibitory control follows a protracted time-course. Although early signs of inhibitory control skills already emerge during the first few years of life, inhibitory control capacities undergo significant development throughout childhood and adolescence (Best & Miller, 2010). Between the age of 5 and 8 years, children show significant improvements in complex inhibition tasks (i.e., tasks that require the inhibition of a pre-potent response and the generation of an alternative response) as new inhibitory control strategies come on line and can be deployed (Carlson, 2005; Gerstadt, Hong, & Diamond, 1994). Improvements in accuracy when performing inhibitory control tasks take place throughout adolescence, likely due to increasing efficiency in inhibitory control, rather than development of new inhibitory control capacities *per se* (Bunge, Dudukovic, Thomason, Vaidya, & Gabrieli, 2002; Romine & Reynolds, 2005).

These distinct developmental trajectories enable us to draw different predictions regarding the development of the ability to integrate causal-moral and intentional-moral evaluations in moral judgment of different situations of harm. First, based on the development of emotional arousal to a victim’s suffering, we would expect pre-schoolers’ moral judgment to be mostly sensitive to the agent’s causal role. With the emergence of ToM capacities, we would expect that these children will increasingly generate intentional-moral evaluations, but only when the situation does not require them to resolve a conflict that necessitates the deployment of inhibitory control resources. Therefore, at around the age of 5 years, children should be able to blame agents who attempt to commit harm but their inhibitory control skills should be insufficiently developed to integrate the agent’s innocent intentions in accidental harm, which requires the inhibition of the causal-moral evaluation. At this age, they should thus not be able to reliably judge accidental harm and should evaluate it more severely than adults. The ability to integrate the moral-intentional evaluation of accidental harm into moral judgment, and thus to generate an intent-based moral judgment when faced with accidental harm, should come online later, along with the protracted development of inhibitory control resources.

What is the evidence in favor of this prediction? The first well-known investigations of the development of intent-based moral judgment were conducted by Piaget (1932). He reported that children were not able to prioritize the agent’s intentions before the age of 7 or 9 years. However, these initial findings have been largely criticized (for a review, see Karniol, 1978). Methodological changes in subsequent studies have allowed developmental psychologists to show that children are indeed able to distinguish intentional from accidental harm between the age of 3 and 5 years, depending on whether the agent’s mental states are presented in a very explicit and salient way, and on how salient and available the information is regarding the outcomes that children have to compare (Baird & Astington, 2004; Hebble, 1971; Nelson, 1980; Nelson-le Gall, 1985; Nobes, Panagiotaki, & Pawson, 2009; Zelazo, Helwig, & Lau, 1996). The fact that increasing the saliency of the agent’s mental state and reducing the saliency of the outcomes enables younger children to take intentions into account in their moral judgments, indirectly favors the hypothesis that ToM and inhibitory control are critical to generate intent-based moral judgment. It is possible that young children are only able to integrate information about intentions in their moral judgments when this information is explicit because it reduces the need for them to use their not yet fully developed ToM competences to make an appropriate judgment. It is also possible that when the consequences are attenuated, absent, or at least kept constant across conditions, the conflict that occurs between the information about the agent's intent and the outcomes of his/her actions is attenuated, which reduces the need to deploy inhibitory skills, making the task possible for young children who have not yet developed these skills.

At 5 years, however, children’s moral judgments are not yet comparable with adults’ moral judgments. Until the age of 7–9 years there appears to be increasingly reliable use of the agent’s intention and a decrease in the dominance of causal computations to judge harmful actions (Cushman et al. 2013; Hebble, 1971; Imamoğlu, 1975; Nobes et al., 2009; Zelazo et al., 1996). For example, Cushman et al. (2013) explored children’s *individual* judgments (that is, judgments that are made about each agent separately instead of comparative judgments where the child is asked to compare two agents) about attempted harm and accidental harm in well-controlled scenarios where the intention to cause harm was not overly explicit. They found that the increasing propensity to favor the agent’s intention in moral judgment was mostly explained by the growing ability to integrate the agent’s innocent intentions, enabling the exculpation of agents that perpetrate accidental harm. In other words, it seems that by the age of 5 years, children are more likely to form appropriate severe moral judgments about those agents who attempt to harm someone than to appropriately exculpate an agent who commits accidental harm; the ability to exculpate agents who cause accidental harm only seems to be reliably achieved around the age of 7–9 years (see also Hebble, 1971, for a similar developmental trend). Even though these findings remain to be replicated using other types of well-controlled stimuli, they are in line with the hypothesis made by our model, and suggest that the integration in moral judgment of an agent’s harmful intention is “online” before the ability to integrate an agent’s harmless intent, which we propose is reliant on more developed inhibitory control resources.

Below, we will overview data from experiments that have explored more directly the role of emotional arousal, ToM, and inhibitory control in the development of moral judgment of harmful situations. Regarding the role of emotional arousal in early moral judgment, Killen, Lynn Mulvey, Richardson, Jampol, and Woodward (2011) have shown that children who endorse cause-based judgments when faced with accidental harm (i.e., who blamed the agent) tend to justify the punishment of the agent more in terms of harm caused to the victim, than their peers who endorse intent-based moral judgment (i.e., who exculpate the agent). While this result suggests that the welfare of the victim is a critical feature for young children’s moral judgments, it does not specify that the emotional arousal to the victim’s suffering is the critical input for the formation of their judgment. A recent study by Decety, Michalska, and Kinzler (2011) sheds more light on this issue. The authors have employed an fMRI task with non-verbal stimuli of harmful situations to assess moral processing in a sample spanning a wide age range. They have also recorded pupillary dilation, an index of general affective arousal, during task performance. During the observation of accidental harm, but not of intentional harm, pupillary dilatation was negatively associated with age, suggesting that arousal to accidental harm selectively decreases with age. Whether this diminished emotional arousal rests upon the development of inhibitory control skills remains to be explored in further studies. Similarly, this study has shown interesting neurodevelopmental changes in structures typically involved in affective saliency (i.e., amygdala and insula); response in these areas decreased with age. Even though these data remain correlational, it suggests that processing moral situations is more dependent upon basic affective responses in younger participants than in older participants.

Regarding the role of ToM capacities in the development of intent-based moral judgment, Baird and Astington (2004) have reported a positive correlation between 4- to 5-year-olds’ performance at the classical False Belief Task (FBT)^2^ and their ability to distinguish two agents performing similar actions based on their good or bad intentions. Killen, Lynn Mulvey, Richardson, Jampol and Woodward (2011) have focussed on children’s judgments of accidental harm and have demonstrated that 3- to 5-year-old children who pass the FBT are more likely than their peers to consider the intention of an agent committing accidental harm as “all right,” and less likely than their peer to consider the punishment of such an agent as acceptable. In other words, ToM abilities, at least as assessed by the FBT, appear to be critical for considering the agent’s intentions and for exculpating the agent who caused accidental harm.

The extant developmental findings reported here are in line with the assumptions made by our model. Developmental findings, however, remain incomplete and at times difficult to interpret. In particular, it is important to keep in mind that the FBT is not sensitive to quantifying individual differences in ToM across a wide developmental age. Children who are older than 5 years typically pass the task, yet as detailed above, the ability to generate a fully mature intent-based moral judgment occurs only around age 7–9 years. We also know that children’s FBT performance is correlated with their inhibitory control resources (Apperly, Riggs, Simpson, Chiavarino, & Samson, 2006; Carlson, Moses, & Claxton, 2004). In order to clarify the relative contributions of ToM and inhibitory control on the development of moral judgment, further studies need to employ ToM tasks that capture individual differences in deploying mentalising computations across different ages and which do not have an inhibitory control element (Apperly et al., 2011; Dumontheil et al. 2010) or need to include additional inhibitory control conditions/tasks to enable researchers to disentangle the contribution of inhibitory control on the reported associations. In addition, the impact of inhibitory control skills on the development of intent-based moral judgment, even though previously suggested (Cushman et al., 2013; Zelazo et al., 1996), has not yet been directly investigated (but see Gvozdic, Moutier, Dupoux, & Buon, 2016, for recent experimental evidence in this direction). Ideally, future studies should investigate the development of intent-based moral judgment together with a direct assessment of all the component processes that are purported to be important for moral computations (i.e., emotional arousal, ToM capacities, and inhibitory control).

Finally, it should be noted that recent studies indicate that infants and toddlers can be sensitive to the agent’s intentions over the agent’s causal role with regard to negative outcomes (Hamlin, 2013; Hamlin, Ullman, Tenenbaum, Goodman, & Baker, 2013; Vaish, Carpenter, & Tomasello, 2010), However, these studies differ from the work with child and adult samples in several ways (Buon et al., sous presse; Margoni & Surian, 2016), which means that it is not possible to directly compare them to these studies. For example, the infant studies have typically depicted scenarios with salient intentional cues and have not included explicit information about the affective outcomes of the actions to the victim (such as distress cues). It is therefore not possible to conclude whether the infant studies have probed infants’ ability to track and evaluate harmful agents or merely uncooperative ones. In most of these studies, participants were shown agents whose actions were performed with the intention to help or hinder another agent to achieve a goal. Even though it might be uncomfortable to not achieve a goal, it is unlikely that this negative outcome is comparable to the physical harm portrayed in the studies with older children or adults described above (see Buon et al., 2014 for a similar argument). Given these differences in the stimuli used to probe infants’ and older children’s evaluative abilities, it is possible that a discrepancy between findings of infants’ and children’s socio-moral competencies reflects the nature and intensity of the conflict induced by the stimuli used in these studies (agent’s intentions are less difficult to deduce in infant studies). Further studies that systematically and parametrically manipulate the salience and nature of the agents’ causal role and intention should shed more light on this issue. Finally, in the infant and toddler studies their social behavior is used as an index of their moral judgment (e.g., grasping one of the two agents presented; helping behavior). While social behavior has been interpreted as a precursor of moral judgment abilities, this assumption remains to be established empirically by exploring longitudinally whether infants’ and toddlers’ early social behaviors predict their moral judgments abilities later on (see Apperly & Butterfill, 2009; Yamaguchi, Kuhlmeier, Wynn, & vanMarle, 2009, for comparable debate regarding early vs. late ToM competencies).

## Insights from clinical populations

We will now turn to clinical populations to explore whether they can provide more insights regarding the processes implicated in the computation of moral judgment of harmful actions.

### The importance of affective processes in moral judgment abilities: Investigating psychopathy and alexithymia

Individuals with psychopathy are of particular interest for investigating the importance of basic emotional processes in the ability to judge harmful actions. Psychopathy is a personality disorder that involves emotional dysfunction, including diminished empathy, reduced guilt, and lack of attachment to other people (Blair, 1995; Viding, McCrory & Seara-Cardoso, 2014). Individuals with psychopathy are substantially more likely than typical individuals to engage in amoral and antisocial behavior. Several decades of experimental research have probed the deficits of individuals with psychopathy and have, for example, documented reduced autonomic responses to the distress of others and reduced recognition of others’ sad and fearful (and possibly other) expressions (Blair, 2013; Dolan & Fullam, 2010). In contrast, individuals with psychopathy do not appear to have impairments in ToM and inhibitory control tasks, as long as these tasks do not require processing of affective content (Blair, Marsh, Finger, Blair, & Luo, 2006; Shamay-Tsoory & Aharon-Peretz, 2007).

Another interesting population for the study of morality are individuals with a high level of alexithymia. Alexithymia is a subclinical personality construct characterized by a reduced capacity to experience emotions, absence of tendency to reflect on one’s own emotions, difficulty in identifying feelings and bodily sensations associated with emotional arousal, and in describing these feelings to other despite a basic awareness of bodily arousal and sensation (Lane, Ahern, Schwartz, & Kaszniak, 1997). At a cognitive level, elevated traits of alexithymia are associated with impaired performance on a number of important social cognitive tasks, including emotion recognition (Cook, Brewer, Shah, & Bird, 2013) and empathy (Bird et al., 2010; Moriguchi et al., 2007).

Even though the nature and the origin of the emotional impairment in these two populations differs in several ways (Bird & Viding, 2014), both individuals with psychopathy and those with high levels of alexithymia have been reported to have impaired emotional arousal to others’ distress, a component which is critical for the ETIC model. According to our model, emotional arousal to a victim’s distress (perceived or imagined) is an important process when making moral evaluations about causal responsibility and harmful intent. Therefore, we predict that both individuals with psychopathy and those with high levels of alexithymia should be impaired at generating moral judgments about accidental and attempted harm. More specifically, individuals with psychopathy and individuals with high levels of alexithymia should judge the agents that have perpetrated accidental or attempted harm less severely than healthy controls. Young, Kruepke, and Newman (2012) have found that individuals with psychopathy are more likely than controls to consider the action of someone committing accidental harm as permissible. Patil and Silani (2014) have also found that people with higher alexithymia scores are more likely to find accidental harm more acceptable than those with lower levels of such traits. These interesting findings suggest that these individuals prioritize the intentional-moral evaluation over the causal-moral evaluation more than controls do, possibly because the victim’s distress cues does not influence their moral judgment.

In contrast, Young et al. (2012) did not find a difference between individuals with psychopathy and typically developing individuals in judging attempted harm (but see Trémolière & Djeriouat, 2016). Similarly, Patil and Silani (2014) do not find that individual differences in alexithymia related to how likely people were to find attempted harm acceptable. These findings are more puzzling to explain and do not appear to be in line with our claim about the role that emotional arousal plays in moral judgment of attempted harm. It is possible that individuals with psychopathy and high alexithymia traits use alternative cognitive strategies to appropriately blame an agent who attempts harm despite their impaired affective response when representing the agent’s intentions. This strategy could rest upon the application of general abstract rules about what is “right” and what is “wrong.” This hypothesis is in line with the idea that individuals with psychopathy may use an alternative cognitive route when processing moral content (Cima, Tonnaer, & Hauser, 2010; Glenn, Raine, Schug, Young, & Hauser, 2009; Tassy, Oullier, Cermolacce, & Wicker, 2009). In other words, it is possible that individuals with high levels of psychopathy or alexithymia are able to represent the content of an agent’s intention (to cause harm) and to use this representation of an intention that is “wrong” and “against societal rules” for the purpose of moral judgment – but without any emotional aversion to this representation. An important avenue for future research would be to explore the ability of individuals with basic emotional arousal impairments to generate moral judgments under different cognitive load conditions. More than allowing us to understand the role of automatic emotional processes in judging harmful intentions, such studies may help us to shed a new light on the dissociation between “knowing” and “caring” that is usually observed in these populations (Cima et al., 2010).

### The importance of Theory of Mind (ToM) capacities in moral judgment abilities: Individuals with high functioning autism (HFA) and Asperger syndrome (AS)

Another clinical population that is of particular interest for the purpose of validating the assumptions made by our model are individuals with high functioning autism (HFA) and Asperger syndrome (AS). These individuals, despite having spared intellectual abilities, unimpaired psychophysiological response to others’ suffering (Blair, 1999; Sigman, Dissanayake, Corona, & Espinosa, 2003), and inhibitory control resources (for review see Russo et al., 2007), have well-documented ToM impairments (Castelli, Frith, Happé, & Frith, 2002; Gilbert, Jones, & Happe, 2010; Happé, 1994). Individuals with HFA/AS (unlike subjects with low functioning autism) usually pass first- and second-order ToM tasks (Bauminger & Kasari, 1999; Happe, 1995), likely using compensatory verbal strategies (Happe, 1995). However, in more advanced ToM tasks, such as the Strange Stories Task or the *Faux Pas* Task,^3^ they typically reveal their difficulties in reasoning about others’ mental states (Baron-Cohen, O’Riordan, Jones, Stone, & Plaisted, 1999; Happé, 1994; Zalla, Sav, Stopin, Ahade, & Leboyer, 2009).

According to our model, given their ToM deficit, individuals with HFA/AS should present difficulties in integrating both harmful and harmless intentions of an agent. Partially in line with these predictions, recent studies have reported that individuals with HFA/AS judge an agent committing *accidental harm* more severely than do healthy comparison individuals (Buon, Dupoux, Jacob, Chaste, & Leboyer, 2013; Moran, Young, Saxe, Lee, O’Young, Mavros, & Gabrieli, 2011). This finding has been reported in two different studies using two different types of stimuli (non-verbal dynamic stimuli and verbal stimuli). Importantly, this impairment seems to relate to basic intentional computation of the agents’ innocent intentions but also extends to the integration of an agent’s intentional cues into moral judgments. Indeed, Buon, Dupoux, et al. (2013), using non-verbal dynamic stimuli, have shown that individuals with HFA/AS are less likely than controls to correctly interpret the agent’s intentions. However, the authors have also demonstrated that even when individuals with HFA/AS are able to report the agent’s intentional states, their judgments about accidental harm are still more severe than that of control participants. These findings suggest that ToM capacities are important for computing basic intentional computations as well as intentional-moral evaluations.

Our model also predicts that individuals with HFA/AS would judge attempted harm less severely than healthy controls. This is because the ETIC model predicts that the intention to harm another person would not be computed and integrated as reliably by individuals with ToM impairments as it would be by individuals with intact ToM abilities. However, contrary to this prediction a recent study reported comparable moral evaluations of attempted harm for HFA/AS and typically developing individuals (Moran et al., 2011). Findings from this study suggest that individuals with HFA/AS are able to (i) make basic intentional computations about the agent’s harmful intent and (ii) evaluate them from a moral standpoint. However, we are not entirely sure that the findings by Moran and colleagues can be interpreted in such a straightforward fashion. In the task used in this study all the elements required to compute the agent’s intentions are verbal and explicit, making the harmful intention of the agent readily accessible – i.e., the computation of intent under these tasks conditions is unlikely to be challenging for individuals with HFA/AS. In addition, once the agent’s harmful intention is successfully computed, integrating this intention into moral judgment is not cognitively taxing as the representation of the agent’s intention does not enter into conflict with the output of the causal evaluation (as in the case for accidental harm). Indeed, it has been proposed that ToM computations/capacities required to properly judge an agent causing accidental harm would need to be more robust than the ones implicated in judging attempted harm (Young & Saxe, 2009). In the case of accidental harm, the representation of the agent’s intention needs to be sustained against the conflict caused by the representation of the agent’s causal role. In case of attempted harm, the agent fails to hurt the intended victim and no actual distress cues or physical outcomes actively conflict with the representation of the agent’s intention. Therefore, one may propose that compensatory mechanisms of individuals with HFA/AS are sufficient to compute and integrate the agent’s harmful intent (particularly if the task format minimizes the requirements for computing intent) but perhaps not enough to generate apparent normal judgment when processing accidental harm. If this hypothesis is correct, one might also expect individuals with low functioning autism or with lower verbal abilities (e.g., younger children with HFA or AS) to be impaired in providing typical moral judgment of situations involving of attempted harm. Another interesting possibility would be that the spared ability to judge attempted harm in individuals with HFA/AS relies on their intact emotional arousal toward others’ suffering. To provide context, it has been proposed that that during childhood, the aversive response that is repeatedly associated with intrinsically harmful acts (e.g., hitting, shooting) leads children to form an automatic response to such actions (Cushman, Gray, Gaffey, & Mendes, 2012). This suggests thus that once this emotional conditioning has taken place, individuals are able to generate negative evaluations to those type of actions without any additional computation. By contrast, blaming an agent who tried to cause harm through an action that did not typically lead to harmful outcomes during the development (e.g., pulling a lever) would require individuals to engage in ToM computations, enabling them to represent the victim’s distress. According to this hypothesis, appropriate judgments of individuals with HFA/AS toward attempted harm should be restricted to familiar harmful actions (i.e., those that have been negatively reinforced during the development).

### The importance of inhibitory control in moral judgment abilities: Need for evidence from populations with inhibitory control deficits

Regarding the importance of inhibitory control resources in moral judgment’s abilities, one population that is of particular interest are individuals with attention deficit/hyperactivity disorder (ADHD). Children with ADHD present symptoms of inattention, hyperactivity, and/or impulsivity (Kuntsi et al., 2014). At the cognitive level, children with ADHD are more likely to present deficits in response inhibition, planning, and cognitive flexibility when compared to control children (Willcutt et al., 2005). Exploring these children’s ability to generate moral judgments could thus help us to understand the importance of inhibitory control in moral judgment abilities. According to our model, a cognitive profile marked by impaired inhibitory control skills should lead to more severe moral judgments of accidental harm: it should be more difficult for these individuals to inhibit their emotional reaction to the harm that is caused to the victim. To date, and to the best of our knowledge, no study has directly explored moral judgment of accidental (or attempted) harm in this population.

### Insights from clinical populations: Summary

Extant findings from clinical populations enabled us to further evaluate our theoretical proposition. Atypical moral judgment of accidental harm seen in individuals with psychopathy and high levels of alexithymia (i.e., who judge less severely agents who commit accidental harm) provides support in favor of the prediction that intact affective processing of distress’ cues may be crucial for the evaluation of an agent’s causal role in effecting harm. The atypical profile of moral judgment seen in individuals with HFA/AS when faced with accidental harm provide evidence in favor of the prediction of our model that ToM capacities may be critical for generating intentional/moral evaluations and for the integration of agents’ innocent intentions into moral judgment. Findings from individuals who have HFA/AS add to the evidence base from fMRI studies, which indicate that brain areas associated with ToM processing, including rTPJ, are implicated in the ability to exculpate an agent for his accidental harm, and with developmental studies that have demonstrated that the ability to pass the false belief task is significantly related to the ability to exculpate an agent who has committed accidental harm. Further empirical research is required to explore whether individuals with psychopathy and HFA/AS use the same cognitive mechanisms as typical individuals when they process attempted harm. Insights from inhibitory disorders, such as ADHD, are currently lacking and could provide an interesting further avenue for testing the model.

## Future directions

In this paper, we presented a model where the ability to generate appropriate moral judgments of harmful actions relies on separable evaluative processes in which emotional arousal to others’ distress, ToM capacities, and inhibitory control resources play a critical role. The findings we reviewed from cognitive psychology, neuroscience, developmental psychology, and clinical populations provide tentative support in favor of the model. However, a number of questions still need to be addressed in future studies to validate the model in a more comprehensive way.

### The role of affective processing in integrating information about the agent’s causal role and intention to harm into moral judgment

In our model, affective processes are proposed to be critical for both the evaluation of the agent’s harmful causal role and the evaluation of the agent’s harmful intent to moral judgment. Specifically, we argue that the emotional arousal triggered by the perception of a victim’s suffering is required to assign blame to an agent for the harm caused. Furthermore, we propose that the same type of affective response is critical for the sensitivity to the agent’s harmful intention by allowing individuals to ‘negatively color’ the representation of the agent’s intention. Importantly, in our model, the negative emotional state that arises from the perception (in case of actual/intentional and accidental harm) or the imagination (in case of attempted harm) of the victim’s suffering provides the critical input for valuing the agent’s moral statue. Findings on adults with psychopathy and those with high levels of alexithymia (i.e., populations with blunted (psychopathy) and/or undifferentiated (alexithymia) emotional responses) suggest that intact processing of others’ distress/suffering is critical when computing the agents’ causal role. Despite these findings in favor of our initial assumptions, further studies are required to understand the precise role of an intact response to distress cues in the moral computation of the agent’s causal role. For instance, distress cues could mainly act as salient features that automatically trigger the individuals’ attention towards the immoral actions and lead individuals to prioritize the agent’s causal role. If the role of emotional arousal is restricted to its role in directing attention (Decety et al., 2011), one should expect emotional response to occur prior to any moral evaluations. If a moral task was embedded with an attentional cuing paradigm to orient individuals’ intention towards the victim’s suffering, one may expect participants to be able to evaluate the agent’s causal role despite blunted or undifferentiated affective response (see Meffert, Gazzola, den Boer, Bartels, & Keysers, 2013, for findings that suggest that this may be the case).

With respect to the ability to integrate the agents’ harmful intent into moral judgments, we found little support in favor of the assumption that emotional arousal in response to (the representation of) others’ suffering is required for judging attempted harm (i.e., both individuals with psychopathy and those with high levels of alexithymia seem to be comparable to healthy controls in judging attempted harm). We reported that vmPFC patients, who are also thought to present affective impairments, do present atypical judgments of attempted harm and seem to be less likely than controls to judge this type of harm as morally wrong (Ciaramelli et al., 2011; Young, Bechara, Tranel et al., 2010). However, given the heterogeneity of the vmPFC’s functions (see summary section Summary: Insights from studies of adult samples) current findings are not sufficiently clear-cut to identify which specific process or processes underlie both typical and atypical pattern of moral judgment of attempted harm in vmPFC patients. In order to understand whether emotional arousal to others’ suffering is critical for generating moral judgment about attempted harm, the first step would be to systematically and more directly explore individuals’ affective response when they judge agents that have attempted to harm. The effect of other manipulations on judgment about attempted harm should also be explored. One may, for instance, wonder if modulating the status of the target of the harmful intent (previous research has demonstrated that people show greater sensitivity for the suffering of in-group compared with out-group members for instance, e.g., Gutsell & Inzlicht, 2012) has an effect on the blame assigned to the perpetrator of the action.

### The role and nature of ToM mechanisms in moral judgments

The second question that should be addressed in future research is the role and nature of the ToM mechanisms in the ability to generate intent-based moral judgments. The results we report above from fMRI, developmental psychology, and clinical studies strongly argue in favor of ToM capacities being important for generating intent-based moral evaluations. However, we also reported counterintuitive findings from a study, which showed that individuals with AS are impaired in moral judgment of accidental harm but not of attempted harm. This allows us to propose two contrastive hypothesis : First, in line with Young & Saxe (2009), that ToM computations need to be more “robust” to integrate moral evaluations of the agents’ innocent intentions than to integrate moral evaluations of agents’ harmful intentions into moral judgment. Second, it may be possible that the need for adults to engage in ToM computations for blaming attempted harm depend on the type of actions performed by the agent. Whereas this later hypothesis allowed us to make precise predictions that may be tested by systematically varying the nature of the actions (i.e., whether the action evaluated have been systematically and repeatedly associated with harmful outcomes during the development, see section The importance of Theory of Mind (ToM) capacities in moral judgment abilities: Individuals with high functioning autism (HFA) and Asperger syndrome (AS), the first hypothesis requires us to consider ToM broadly, with regards to all the facets this concept may reflect.

Indeed, as outlined by Apperly (2012), ToM refers to a set of representations, conditions and cognitive processes (e.g. inhibitory control, language, memory) that allow people to compute what others are thinking during everyday interactions. ToM capacities also relate to the conceptual understanding of the key notions of desires, intentions, knowledge and beliefs, as well as the way in which these concepts are interrelated – likely a result of a developmental history of making computations of other people’s mental states. Finally, there can be substantial individual differences in how motivated people are to mentalize about other people’s desires and beliefs (i.e., trait-like tendency for paying attention to or caring about what others think; Meins & Fernyhough, 1999). Thus, it will be of interest to assess which aspects of ToM capacities account for: (i) inter-individual differences in ToM reasoning in relation to moral judgments; and (ii) the differential involvement of ToM capacities in the integration of agents’ harmful and innocent intents. For example, do individual differences in judgment of accidental harm stem from individual differences in the motivation to use ToM to explain others behavior, from individual differences in the flexible use of ToM concepts, or perhaps from a combination of the two? We hypothesize that ToM capacities need to be more “robust” in moral judgment of accidental harm than in moral judgment of attempted harm. The notion of robustness, however, remains to be defined and future work should explore more precisely whether integrating the agent’s harmful and harmless intentions rely on distinct conceptual tools, yield different processing costs, and/or are potentially differentially influenced by motivational factors. Further studies using paradigms that assess different aspects of ToM and moral judgment tasks simultaneously are thus needed to clearly isolate the role of ToM capacities in moral judgments of typical adults as well as individuals with autism.

### Exploring the contribution of inhibitory control resources

Evidence in favor of the importance of inhibitory control resources is relatively limited and remains indirect (Treadway et al., 2014; Young et al., 2007). Future studies need to directly investigate the impact of these skills on moral judgment computations. Providing a direct association between inhibitory control skills and lenient judgment about accidental harm would argue in favor of the ETIC model of morality. Future work in experimental psychology should also directly manipulate inhibitory control resources (Houdé & Borst, 2014) to explore their impact on moral decision making (see for instance Gvozdic et al., 2016). However, given the important contribution of inhibitory control skills in the subcomponents of the ETIC model (and especially ToM capacities; Apperly & Butterfill, 2009; Carlson et al., 2004), this would not be sufficient to demonstrate that inhibitory control contributes actively and selectively to override the prepotent response triggered by the perception of agent’s harmful causal role. For instance, without controlling for the ability to detect the agent’s intention, one may argue that inhibitory control skills are required to disengage individuals from the processing of the harmful outcomes, allowing them to engage in computations about the agent’s intention (i.e., inhibition would be required *before* any basic intentional computation and would be required to properly detect the agent’s intentions). Another plausible scenario would be that inhibitory control resources are only required to accomplish complex and sophisticated ToM computations. According to these two hypothesis, *active* inhibition may not be required to provide a lenient moral judgment of agent that caused accidental harm. In those cases, one may argue that the inhibition of the prepotent cause-based response would operate *indirectly* via the selective activation of the relevant content (i.e, the agent’s mental state) and not the suppression of the irrelevant one (i.e., the agent’s causal role). This kind of “passive inhibition” (Hofmann, Schmeichel, & Baddeley, 2012) would predict inhibitory control to predict ToM computations only but not lenient moral judgment about accidental harm.

In sum, future work may need to take into consideration how and when inhibitory control resources operate on the generation of moral judgment by systematically controlling for all the abilities and operations in moral judgment that may involve inhibitory control resources.

### Towards an ecologically valid ETIC model of morality

A recurring issue in the field of moral cognition is that of ecological validity. When we witness actual intentional, accidental or attempted harm, we are likely to have a negative emotional response, to deploy our ToM competencies and/or inhibitory control to understand the situation perceived and evaluate the agents involved. However, we may wonder whether those process are engaged similarly in real life events and in extremely controlled but features-limited stimuli used in experimental settings (Pan & Slater, 2011; Patil, Cogoni, Zangrando, Chittaro, & Silani, 2014). For instance, Pan and Slater (2011) investigated whether adults’ moral judgment in Trolley-like dilemmas were similar in classical experimental settings and virtual reality settings. Preliminary results showed that although participants in virtual reality settings presented similar responses to moral dilemmas as they did in a previous online survey, participants that were placed in an immersive virtual environment were more likely to commit mistakes (e.g., impulsively pressing a wrong button) than those that did the experiment in a desktop with virtual reality. However, those who were placed in immersive virtual reality also presented a higher proportion of utilitarian judgments in a questionnaire with similar dilemmas that followed the virtual reality conditions. While the reasons for this differential pattern of response remain to be investigated, these findings might suggest that individuals experience stronger emotional responses when making decisions in more real-life environments, thus causing them to act more impulsively and make more ‘mistakes,’ whilst at the same time influencing them to generate more ‘rational’ responses afterwards. Applied to the integration of information about intention, we may wonder whether individuals would be more or less sensitive to information about intentions in their everyday decision making. On the one hand, it is possible that in real life conditions, people are more likely to be aroused by the harm caused, making them less likely to integrate information about intentions in their appraisal of the situation. On the other hand, it is possible that people may generate more intent-based judgments when facing situations similar to ones experienced before. Indeed, we have to acknowledge that the ETIC model and the current review are based on the extant empirical literature, which is largely restricted to very controlled experimental protocols. It remains to be seen how well the model can serve in accounting for every day, naturalistic moral judgments.

Importantly, in real life, determining the intentional structure of a given action is likely to rely on many more features that the the strictly controlled ones we have focused on. In this article, we mostly focused on whether individuals successfully rely on high order ToM reasoning to infer others’ complex mental states (such as desires, believes, and false believes) and generate intent-based moral judgment. However, in real life, features such as the goal-directedness of the action performed, its controllability (Weiner, 1995), or the agents’s emotional states (e.g., whether she/he is surprised or happy about the results of her/his action; Behne, Carpenter and Tomasello, 2005) may be critical in determining saliency and helping individuals to determine whether someone acts intentionally or not. Since most of the critical features that feed into these decisions are processed using lower-level mechanism that are available early during the development (Brandone & Wellman, 2009; Cannon & Woodward 2012), it is possible that information about agents’ intentions are incorporated much more easily in daily-life settings than in the experimental settings.

Although evaluating the intentions of an agent may be sufficient for exculpating the agent of an accidental harm in experimental settings or in daily-life interactions, recent developmental studies have also highlighted the relevance of information about negligence in individuals’ tendency to blame an agent for his accidental harm (Nobes et al., 2009). The ability to process carelessness is probably highly dependent on the integrity of ToM capacities, since it requires the integration of information about the agent’s knowledge and contextual information, as well as information about the agent’s intentions (Nuñez, Laurent & Gray, 2014). However, attributing carelessness may also depend on individuals’ personality and thus does not exclusively depend on the integrity of ToM capacities. Studies that have explored the processes underlying typical and clinical populations’ sensitivity to information about negligence in morality are still rare but would be be essential to refine the structure of the ETIC model and our understanding of the ability to generate intent-based moral judgments.

### From moral judgment abilities to moral behavior

Another challenge for future research is to systematically investigate the associations between different sub-components of the ETIC model (i.e., emotional arousal, ToM, and inhibitory control), moral judgment abilities and moral behavior. It is unlikely that different types of moral judgment impairments (resulting from atypical emotional arousal, ToM, or inhibitory control processes) all impact similarly on moral behavior.

We can illustrate this with the dissociation between the processes implicated in causal and intentional evaluations and how distinct pattern of compromised processing might result in specific vulnerabilities to proactive and reactive aggression separately. Notably, atypical processing of the negative outcomes following a harmful action (i.e., positive outcome expectancies: expecting less negative outcomes following an aggressive act) is associated with higher levels of *proactive aggression* in individuals (Crick & Dodge, 1996). By contrast, atypical processing of innocent intentions (i.e., misjudging any provocations as guided by malevolent intentions) has been associated with higher levels of *reactive aggression* (Crick & Dodge, 1996). The positive outcome expectancies could be (at least partially) related to a diminished response to others’ suffering while the atypical processing of other’s intentions as malevolent may be related to ToM and/or inhibitory control deficits. Individuals with antisocial behavior disorders are a heterogeneous group: whereas some of these individuals – comparable to individuals with psychopathy presented above – are characterized by diminished response to others’ suffering and positive outcome expectancies following aggression (Blair, 2013; Jones, Happé, Gilbert, Burnett, & Viding, 2010; Pardini, Lochman, & Frick, 2003), others display a high baseline emotional reactivity to salient/provocative stimuli and to the distress of others (Hodsoll, Lavie, & Viding 2014; Kimonis, Frick, Munoz, & Aucoin, 2008; Loney, Frick, Clements, Ellis, & Kerlin, 2010), a tendency to attribute malevolent intention in situations that others’ view more neutrally (Frick & Morris, 2004; Pardini et al., 2003), and poor inhibitory control skills at least in tasks that require control of affective responses (Blair, 2013). No study, to the best of our knowledge has yet systematically explored how the processing atypicalities seen in different types of individuals with antisocial disorders may relate to selective moral judgment impairments. It would be of interest to test whether, for example, evaluating the outcome of aggression positively is linked to a lack of sensitivity to the agent’s causal role or whether the tendency to attribute malevolent intent is related to a diminished integration of the agent’s innocent intention into moral judgment. The ETIC model could, therefore, be applied to systematically extend the current evidence base on antisocial behavior disorders and provide a way of interrogating the relationship between moral judgment and moral behavior.

## Conclusion

Findings in the fields of cognitive psychology and cognitive neuroscience support the notion that moral judgments are contingent on complex and multiple cognitive processes such as emotional arousal, ToM, and inhibitory control. With the ETIC model we attempt to outline the processes (and interactions between the processes) that may underlie our ability to generate moral judgments of basic harmful actions. We reviewed recent findings from cognitive experimental, neuroscience, developmental, and clinical studies, and found important support in favor of the main assumptions of the ETIC model in adults and developmental and clinical populations.

Nevertheless, a number of points of the model remain to be addressed and further work needs to be done in order to extend the evaluation of the ETIC model to real life contexts, as well as to understand the precise nature and role of emotional processes, ToM, and inhibitory control resources in our moral judgment abilities.

In order to achieve this understanding, we propose that future studies should systematically assess different types of moral judgments in relation to the target mechanisms that are proposed to contribute to moral cognition. This could be done in several ways, for example by using electrophysiological measures to index emotional arousal, by employing more sensitive and controlled tasks than the False Belief Task for quantifying ToM capacities, and by including comprehensive measures of inhibitory control resources. Ideally, these measures should be used together to control for the potential overlap that may exist between these cognitive mechanisms. Ultimately, such studies would have the potential to help us to demonstrate the interactive nature of the different evaluative systems that constitute our moral judgment abilities. These types of experiments should be conducted with adults but also with typically developing children and children with different developmental disorders, using longitudinal designs. This should give us a more precise and less speculative picture of the role of, and the nature of, the interaction between empathic processes, ToM abilities, and inhibitory control resources required to develop moral judgment abilities. Additionally, when assessing children of different ages and various clinical groups, measurement of emotional arousal, ToM, and inhibitory control may help explain heterogeneity in moral judgment ability across typical development and within a given pathology.

Future work should also study the importance of those processes for different types of moral judgments separately (e.g., judgment of wrongness, judgment of punishment). Since few studies have systematically compared different types of moral judgments (but see Cushman, 2008; Cushman et al., 2013), we consider that available evidence is presently too scarce to conduct a proper evaluation of the differential involvement of emotional arousal, ToM, and inhibitory control in these different types of moral judgments.

Subsequent empirical studies should employ paradigms that enable researchers to directly contrast predictions made by the ETIC model and Cushman’s model, particularly in relation to developmental samples. The ETIC model proposes that the abilitity to generate intent-based moral judgments mainly relies on the maturation of ToM capacities and inhibitory control resources wheras Cushman’s model posits that the ability to generate intent-based moral judgments largely relies on children’s conceptual development (Cushman et al., 2013). A systematic empirical investigation of the predictions from these two models will arbitrate whether development of moral competencies exclusively relies on the functional integration of multiple cognitive systems and/or the acquisition of specific conceptual knowledge.

Finally, there is a dearth of studies investigating the links between moral judgments abilities and moral behavior. Such studies should come with a full assessment of antisocial behavior disorder symptoms that would include both the severity as well as the type of behavioral problems displayed by participants. This would be essential to understand the links between “basic” cognitive components, moral judgment abilities, and moral behavior as well as the different paths that lead individuals to behave in a morally inappropriate way.

## Footnotes

1 We used the term conflict when both systems are “busy” (when there is a causal analysis to be performed, i.e., a harm has been caused) as well as an intentional state to be processed. In contrast, we used the word competition when only one system is busy. In this case, there is no conflict between the two systems but there is still a competition for the resources involved.

2 In the most common version of the FBT, children are shown a story involving two characters. For example, the child is shown two dolls, Sally and Anne, who have a basket and a box, respectively. Sally also has a marble, which she places in her basket, and then leaves the room. While she is out of the room, Anne takes the marble from the basket and puts it in the box. Sally returns, and the child is then asked where Sally will look for the marble. The child passes the task if she answers that Sally will look for the marble in the basket where she put it; the child fails the task if she answers that Sally will look in the box, where the child knows the marble is hidden, even though Sally cannot know this, since she did not see it hidden there. To pass the task, the child must be able to understand that another person’s mental representation of a situation can be different from his or her own, and the child must be able to predict behaviour based on that understanding.

3 In the Strange Stories task, participants typically have to identify complex social situations of bluff/double bluff, irony. In the Faux Pas task, people have to recognize and explain a Faux Pas, a socially awkward or tactless act, especially one that violates accepted social norms, standard customs, or the rules of etiquette.
